# Awareness and Utilization of HIV Testing and Prevention Services Among Female Sex Workers in Dnipro, Ukraine: Implications for Prevention Program Strengthening From the Dynamics Study

**DOI:** 10.3389/frph.2022.879191

**Published:** 2022-06-10

**Authors:** Joel Derksen, Daria Pavlova, Leigh M. McClarty, Olga Balakireva, Nicole Herpai, Lisa Lazarus, Aruni Tennakoon, Tatiana Tarasova, Robert Lorway, Michael Pickles, Sharmistha Mishra, Souradet Y. Shaw, Marissa L. Becker

**Affiliations:** ^1^Max Rady College of Medicine, Rady Faculty of Health Sciences, University of Manitoba, Winnipeg, MB, Canada; ^2^Ukrainian Institute for Social Research After Olexander Yaremenko, Kyiv, Ukraine; ^3^Institute for Global Public Health, Rady Faculty of Health Sciences, University of Manitoba, Winnipeg, MB, Canada; ^4^Institute for Economics and Forecasting, Ukrainian National Academy of Sciences, Kyiv, Ukraine; ^5^Medical Research Council Centre for Global Infectious Disease Analysis, School of Public Health, Imperial College London, London, United Kingdom; ^6^MAP Centre for Urban Health Solutions, Li Ka Shing Knowledge Institute, St. Michael's Hospital, Toronto, ON, Canada; ^7^Department of Medicine, University of Toronto, Toronto, ON, Canada; ^8^Institute of Medical Sciences, University of Toronto, Toronto, ON, Canada; ^9^Institute of Health Policy, Management and Evaluation, University of Toronto, Toronto, ON, Canada

**Keywords:** sex workers, Ukraine, HIV testing, HIV prevention, non-governmental organizations

## Abstract

**Introduction:**

Approximately 240,000 people live with HIV in Ukraine, concentrated among key populations, including sex workers. Non-governmental organizations (NGOs) play an important role in the funding and delivery of HIV testing and prevention services in Ukraine. These services are set within the context of national healthcare reforms as well as ongoing armed conflict. This study seeks to describe and understand the usage of HIV testing and prevention services among sex workers in the eastern Ukrainian city of Dnipro.

**Methods:**

A cross-sectional bio-behavioral survey was administered in September 2017-March 2018 among 560 sex workers working in Dnipro. Descriptive analyses of survey data are presented alongside multivariable logistic regression models identifying factors associated with NGO awareness and HIV testing in the past 12 months; adjusted odds ratios (AOR) and 95% confidence intervals (95% CI) are reported.

**Results:**

Sixty-two percent of respondents were aware of NGOs offering HIV services. Sixty-eight percent had tested for HIV in the past 12 months, and 51% of those who reported the location of their most recent test were tested at an NGO. Those with 5–9 years in sex work had greater odds of being aware of NGOs (AOR = 5.5, 95%CI: 3.2–9.7) and testing for HIV (AOR = 3.4, 95%CI: 2.0–6.0) compared to those new to the profession. Contact with outreach workers was strongly associated with increased odds of testing (AOR = 13.0, 95%CI: 7.0–24.0). Sex workers in “offices” (brothel-like venues) reported higher odds of testing than all other workplaces, while those in entertainment venues (AOR = 0.3, 95%CI: 0.2–0.5) and public places (AOR = 0.2, 95%CI: 0.1–0.3) reported lower rates. Receiving prevention services, such as free condoms, was associated with increased testing (AOR = 16.9, 95%CI: 9.7–29.3).

**Discussion:**

NGOs in Dnipro, Ukraine play an important role in HIV testing and prevention for women involved in sex work. However, focused efforts should be placed on supporting access to these services for women that are newer to sex work, and those working in entertainment venues or public places. Outreach workers appear to support access to HIV prevention information and supplies and facilitate linkages to HIV testing for sex workers.

## Introduction

Ukraine has the second largest HIV epidemic in Eastern Europe and Central Asia, with an estimated 240,000 people living with HIV, or 1% of the general population (1). The HIV prevalence is unevenly distributed among key populations, including people who inject drugs, men who have sex with men, and sex workers (1). As of 2019, there were an estimated 86,600 sex workers in Ukraine, with an HIV prevalence of 5.2%, more than five times that of the wider population (2). Early diagnosis and treatment remain a challenge in Ukraine, with only 56% of people living with HIV being aware of their status (3). Routine testing is a critical component of a robust HIV prevention strategy; however, its population-level impact is dependent upon the availability, accessibility, and utilization of testing services (4). During 2020, 33 non-governmental organizations (NGOs), operating primarily in large cities, provided HIV and sexually transmitted infection (STI) prevention services to sex workers in all regions of Ukraine. These services included counseling, condoms, lubricant distribution, HIV and syphilis testing, and early tuberculosis detection (5).

In Ukraine, HIV testing and prevention services for sex workers are available through both governmental and non-governmental platforms, although a lack of coordination between state-run services and NGOs continues to be a challenge (6). Historically, international donors and local NGOs have played a vital role in providing and funding these services due to a lack of government resources directed toward testing and prevention. This pattern persists, despite attempts to move toward increasing government responsibility for funding and implementation of these programs (6). While NGOs are instrumental in testing and prevention, HIV treatment is organized, operated, and provided by state-funded “AIDS centres” (7). Since 2017, Ukraine has been in the process of implementing healthcare reform to improve funding, management, and operationalization of the healthcare system more broadly. This includes a goal to transition HIV prevention services from donor to government funding over time; however, as of 2020, only about half of the funding for HIV came from the government (8).

While HIV testing and prevention services are available across the country, sex work in Ukraine operates in a criminalized and highly stigmatized context. As in many countries, both HIV and sex work bear a high level of stigma and discrimination in Ukraine, affecting sex workers' ability to safely access testing and prevention services (6, 9–11). This stigma is widely present in societal discourse, and is further fueled by administrative and criminal laws prohibiting sex work (10). Past attempts to change these policies have been met with deep resistance from government and civil society (10). However, discussions continue, as recent studies in Ukraine link the criminalization of sex work and a growing epidemic of HIV and other STIs, as well as experiences of violence and civil rights violations (12). In addition, Ukraine has endured ongoing armed conflict in the eastern Donbas region since 2014, which started following the invasion and subsequent annexation of Crimea in the same year. More recently, in the early hours of 24 February 2022, Russian aggression in the Donbas was acutely exacerbated and has since expanded to a full-scale, nation-wide war. Armed conflict creates and sustains complex humanitarian crises, such as intensified poverty, increased migration and displacement, and weakened health systems that have particular implications for existing epidemics, including HIV (13–15). Research has demonstrated a need to improve the availability and accessibility of HIV prevention and care services for sex workers in conflict-affected areas, with an identified gap in the literature for understanding the experiences of conflict-affected sex workers in accessing these services (16). Within the scope of the larger *Dynamics* study examining the impact of conflict on the dynamics of sex work and the HIV epidemic in eastern Ukraine (17), this paper seeks to describe and understand the utilization of HIV testing and prevention services among sex workers in the eastern city of Dnipro. It is of particular importance to note that all data collection and analyses presented here were completed prior to the escalation of the ongoing war and consequent humanitarian crisis in Ukraine.

## Methods

### Study Setting

Dnipro is a city of ~1 million people located in eastern Ukraine (18). It is situated approximately 200 km from the conflict zone in the Donbas region, and as such, has become a common destination for internally displaced persons (19). Given its relatively close proximity to the conflict zone, along with a high prevalence of HIV among sex workers (6.7% as of 2014) (20) and an estimated sex worker population of 1,087 (21), Dnipro was determined to be an important location to situate the *Dynamics* study (17). The study was conducted in partnership with the Ukrainian Institute for Social Research and The Center for Public Health of the Ministry of Health of Ukraine.

### Data Collection

Data were collected from September 2017 to March 2018 through a cross-sectional bio-behavioral survey administered to a sample of 560 sex workers in Dnipro. Sample size was determined based on 80% power and a 5% error in the outcome of the experience of physical violence among sex workers during encounters with clients (current level 42%) (20, 22). Female sex workers aged 14 years or older who self-identified as having engaged in sex work for at least the past 3 months, were recruited by outreach workers at selected places associated with sex work (“hotspots”). Prior to participant recruitment, comprehensive geographic mapping of hotspots and population size estimations were conducted, following previously described procedures (17), to select a representative sample of hotspots. Within each hotspot, a random selection of sex workers were approached and eligible participants who were interested completed a face-to-face, structured questionnaire administered by a trained interviewer in a mobile van on site, following written, informed consent. Data collection continued until the target sample of 560 participants had been interviewed, facilitated by the close cooperation of social mobilizers, who communicated the importance and relevance of the study to participants. Survey questions were informed by Integrated Biological and Behavioral Survey Guidelines (23), as well as other sex worker surveys, including the *Transitions* study (24), an earlier study piloted and locally validated in Dnipro. Participants were asked questions about basic socio-demographics, experiences of sex work, access to HIV prevention and care programs and related services, and HIV testing behaviors. Following the questionnaire, participants who provided consent gave a finger poke blood sample for rapid HIV testing (SD Bioline HIV 1/2 3.0, Wantai Rapid Test for Antibody to HIV 1+2, and Profitest Rapid Anti-HIV 1/2 Test) and HCV testing (Wantai Rapid Test for Antibody to HCV) along with pre- and post-test counseling. Dried blood spot (DBS) samples were performed for confirmatory serology. Findings from biological data will be published separately.

### Ethics Approval and Consent to Participate

Ethical approval was obtained from the Health Research Ethics Board at the University of Manitoba, Canada [HS20653(H2017:097)]; the Ethical Review Committee of the Sociological Association of Ukraine, and the Committee on Medical Ethics of the L. Gromashevsky Institute of Epidemiology and Infectious Diseases at the National Academy of Medical Sciences of Ukraine, Kyiv. All participants provided written, informed consent. Participants 14 years of age were considered mature minors and provided consent to take part in the study without consent from their parent or guardian. This was approved by the local ethics committees.

### Variable Definitions

Selection of key variables for analyses was based on the framework developed for the *Dynamics* study (17) and other relevant frameworks in the literature (9, 25–27).

### Dependent Variables

*Awareness of NGOs* was determined by asking, “Are you aware of any organizations working on the prevention of HIV/AIDS for sex workers in Dnipro?” Two survey questions were combined to derive the binary variable “*Tested for HIV in the past 12 months*.” First, all participants were asked whether they had *ever* been tested for HIV. For those who had ever been tested, a subsequent question asked, “How many times did you test for HIV in the past year?” Participants who responded with ≥1 tests were categorized as “Yes,” regardless of whether they reported a previous positive HIV test. Participants who indicated that: they had *never* received an HIV test; responded “Yes” to *ever* having had an HIV test but reported 0 HIV tests in the past 12 months; and any participant who could not be certain about HIV tests, specifically, were all categorized as “No.”

### Independent Variables

*Years in sex work* was calculated as the difference between a sex worker's age at time of survey and their age when they first self-identified as a sex worker. *Education* referred to the highest level of formal education or training completed by participants. *Most common workplace* was defined as the most common meeting place where a sex worker connected with their clients, and has previously been described in detail (28). *Offices* (or brothels) were understood to be establishments owned and operated by one or more manager(s) who employ sex workers. *Apartments* included one or more women working out of an apartment which they might own or rent and where they may or may not also reside. *Entertainment venues* included nightclubs, casinos, restaurants, bars, art clubs, and strip bars, while public places included open-air spaces such as streets or parks. *Number of clients, past 30 days* referred to the number of different male clients with whom a sex worker reported having sex in the past 30 days. To assess the *influence of conflict on entry into sex work*, respondents who entered sex work after the conflict began in 2014 were asked, “Do you think the conflict played a role in your decision to enter sex work?” The *recent migration* variable was defined as having lived in more than one city, for at least 3 months each, since the beginning of the conflict in 2014.

A client was determined to have been *registered with an NGO* if they reported to be clients of any NGO providing HIV prevention services (i.e., possessing a registration card or having been assigned a unique registration code). Participants were determined to have been *contacted by an NGO* if they had been contacted, ≥1 time in the past 6 months, by a social worker or outreach worker employed by an NGO providing HIV prevention services. The *awareness of NGO and receipt of outreach services* variable was generated combining the previously defined *awareness of NGO* and *contacted by outreach* variables. *Awareness of NGOs* serves as both the outcome in multivariable logistic regression models and as a component of an independent indexed variable in models with HIV testing as the outcome variable. In Dnipro, HIV prevention services provided through NGOs are delivered solely through outreach. As such, sex workers may not identify outreach workers as being affiliated with a particular NGO, and thus may be contacted and receive services without reporting awareness of specific NGOs. As has been noted in previous work in Ukraine (29), being registered with an NGO is not a prerequisite for receiving prevention services. Finally, sex workers' uptake of HIV prevention services from NGOs in Dnipro (including *receipt of condoms, information booklets or brochures on HIV*, and/or *HIV prevention counseling services*) was assessed by asking participants, “Did you use these services in the last 6 months?”

### Data Analysis

Data were analyzed descriptively, and frequencies for measures of NGO service utilization and HIV testing are presented. Multivariable logistic regression models were used to identify factors associated with awareness of services and HIV testing. Awareness of NGOs offering HIV services and HIV testing in the past 12 months were defined as dichotomous outcome measures in two separate models. For each outcome, individual models were constructed to quantify associations with independent variables. Number of years in sex work was included as a control variable in the awareness model while number of years in sex work and previous pregnancy were included as control variables in the HIV testing model, as regular HIV testing is provided as a component of routine prenatal care in Ukraine (30). Crude and adjusted odds ratios (AOR) were calculated and are presented with 95% confidence intervals (95%CI). Participants responding with “I prefer not to answer” were excluded from analyses for those particular variables. All statistical analyses were performed using SAS 9.4 (Cary, NC).

## Results

### Sociodemographic Characteristics

Participants' sociodemographic characteristics, disaggregated by outcome of interest, are presented in Tables 1, 2. In total, 560 sex workers responded to the survey, with a median age of 26 (IQR = 22–30). Thirty-eight percent (*n* = 211) had been in sex work for 5–9 years, and 44% (*n* = 242) were involved for <5 years. Offices were the most common primary workplace for sex work (40%, *n* = 224), with apartments (27%, *n* = 153) and entertainment venues (17%, *n* = 93) being the next most frequent. Forty-two percent (*n* = 237) of respondents had 20–29 clients in the past 30 days, while 31% (*n* = 174) had <20 and 26% (*n* = 148) had 30 or more. Thirty-five percent (*n* = 196) entered sex work after the conflict began in 2014, and 19% of those (*n* = 32/196) reported that the conflict influenced their decision to enter sex work.

**Table 1:** Socio-demographic characteristics of sex worker participants, by NGO awareness (*N* = 560).

	**Aware of non-governmental organization providing HIV prevention services for sex workers**				**Total**	
	**Yes (*n =* 345)**		**No (*n =* 215)**			
	***N***	**%**	***n***	**%**	***N***	**%**
**Age (years)**						
14–18	8	2.32	7	3.26	15	2.68
19–24	123	35.65	108	50.23	231	41.25
25–29	105	30.43	53	24.65	158	28.21
30–34	54	15.65	23	10.70	77	13.75
≥35	55	15.94	24	11.16	79	14.11
**Education**						
Incomplete secondary education	24	6.96	15	6.98	39	6.96
Completed secondary education	119	34.49	91	42.33	210	37.50
Completed post-secondary education	202	58.55	109	50.70	311	55.54
**Years in sex work**						
0–1	26	7.54	52	24.19	78	13.93
2–4	89	25.80	75	34.88	164	29.29
5–9	155	44.93	56	26.05	211	37.68
≥10	75	21.74	31	14.42	106	18.93
Prefer not to answer	0	0.0	1	0.47	1	0.18
**Most common workplace**						
Office	144	41.74	80	37.21	224	40.00
Apartment	130	37.68	23	10.70	153	27.32
Entertainment venues	26	7.54	67	31.16	93	16.61
Public places	10	2.90	27	12.56	37	6.61
Highways or truck stops	19	5.51	11	5.12	30	5.36
Other	16	4.64	7	3.26	23	4.11
**Conflict influenced entry into sex work**						
No	80	23.19	71	33.02	151	26.96
Yes	10	2.90	22	10.23	32	5.71
Prefer not to answer	5	1.45	8	3.72	13	2.32
Entered sex work before conflict began	250	72.46	114	53.02	364	65.00
**Recent migration**						
No	307	88.99	182	84.65	489	87.32
Yes	38	11.01	33	15.35	71	12.68

**Table 2:** Socio-demographic characteristics of sex worker participants, by HIV testing in the past 12 months (*N* = 560).

	**Tested for HIV in the past 12 months**				**Total**	
	**Yes (*n =* 379)**		**No (*n =* 181)**			
	***n***	**%**	***n***	**%**	***N***	**%**
**Age (years)**						
14–18	8	2.11	7	3.26	15	2.68
19–24	145	38.26	108	50.23	231	41.25
25–29	122	32.19	53	24.65	158	28.21
30–34	55	14.51	23	10.7	77	13.75
≥35	49	12.93	24	11.16	79	14.11
**Education**						
Incomplete secondary education	23	6.07	16	8.84	39	6.96
Completed secondary education	140	36.94	70	38.67	210	37.50
Completed post-secondary education	216	56.99	95	52.49	311	55.54
**Previous pregnancy**						
No	184	48.55	109	60.22	293	52.32
Yes	195	51.45	72	39.78	267	47.68
**Years in sex work**						
0–1	41	10.82	37	20.44	78	13.93
2–4	97	25.59	67	37.02	164	29.29
5–9	167	44.06	44	24.31	211	37.68
≥10	74	19.53	32	17.68	106	18.93
Prefer not to answer	0	0.00	1	0.55	1	0.18
**Most common workplace**						
Office	177	46.70	47	25.97	224	40.00
Apartment	114	30.08	39	21.55	153	27.32
Entertainment venues	45	11.87	48	26.52	93	16.61
Public places	13	3.43	24	13.26	37	6.61
Highways or truck stops	19	5.01	11	6.08	30	5.36
Other	11	2.90	12	6.63	23	4.11
**Number of clients, past 30 days**						
1–19	110	29.02	64	35.36	174	31.07
20–29	172	45.38	66	36.46	238	42.50
≥30	96	25.33	51	28.18	147	26.25
Prefer not to answer	1	0.26	0	0.00	1	0.18
**Conflict influenced entry into sex work**						
No	92	24.27	59	32.60	151	26.96
Yes	13	3.43	19	10.50	32	5.71
Prefer not to answer	3	0.79	10	5.52	13	2.32
Entered sex work before conflict began	271	71.50	93	51.38	364	65.00
**Recent migration**						
No	339	89.45	150	82.87	489	87.32
Yes	40	10.55	31	17.13	71	12.68
**Awareness of NGO and receipt of outreach services**						
Unaware	110	29.02	105	58.01	215	38.39
Aware with outreach	229	60.42	14	7.73	243	43.39
Aware without outreach	40	10.55	62	34.25	102	18.21
**Received condoms in past 6 months**						
No	131	34.56	163	90.06	294	52.50
Yes	248	65.44	18	9.94	266	47.50
**Received HIV booklets/brochures in past 6 months**						
No	182	48.02	168	92.82	350	62.50
Yes	197	51.98	13	7.18	210	37.50
**Received HIV prevention counseling in past 6 months**						
No	227	59.89	174	96.13	401	71.61
Yes	152	40.11	7	3.87	159	28.39

### NGO Service Use and HIV Testing Behaviors

**Figure 1:**
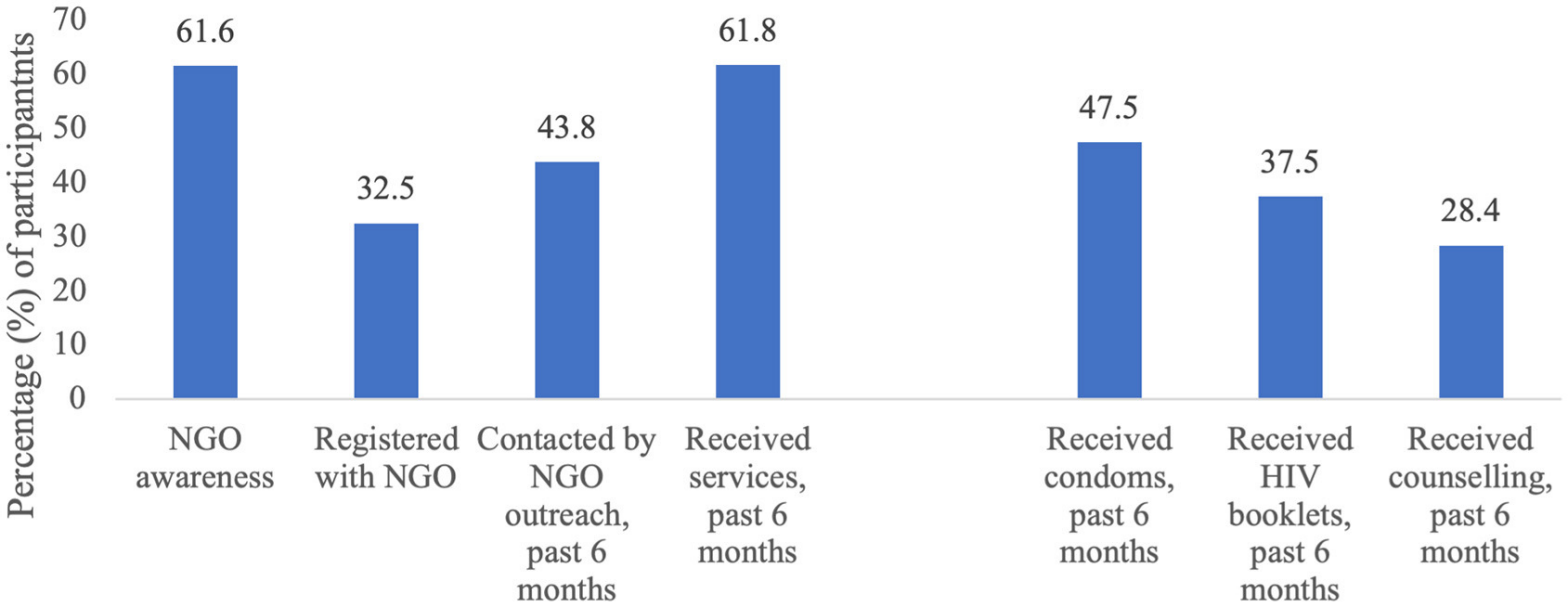
Reported awareness of non-governmental organizations offering HIV prevention services and service use among sex worker participants (*N* = 560).

Sixty-two percent (*n* = 345) of the 560 respondents were aware of NGOs offering HIV prevention services to sex workers. Thirty-three percent (*n* = 182) of sex workers were registered clients of an NGO and 43% (*n* = 243) of respondents had been contacted by an NGO outreach worker in the past 6 months. Receipt of services in the past 6 months was reported by 62% of sex workers (*n* = 346). In the past 6 months, 48% (*n* = 266) obtained condoms from an NGO, 38% (*n* = 210) obtained informational booklets, and 28% (*n* = 159) received counseling on HIV/STI prevention (Figure 1). Sixty-eight percent (*n* = 379) of respondents had tested for HIV in the past 12 months, while 84% (*n* = 472) reported ever testing for HIV. Fifty-one percent (*n* = 233) among those who reported the location of their most recent test (*n* = 461) were last tested at an NGO, while 24% (*n* = 111) tested at a government clinic, 21% (*n* = 98) tested at a private clinic, and 4% (*n* = 19) tested elsewhere.

### Factors Influencing NGO Awareness

Table 3 presents the results from multivariable logistic regression models that assessed associations with awareness of NGOs providing HIV services. More years in sex work was associated with increased NGO awareness, particularly for those in sex work for 5–9 years (OR = 5.5, 95%CI: 3.2–9.7) and those with at least 10 years (OR = 4.8, 95%CI: 2.6–9.1) of experience. Working from an apartment was associated with increased awareness (AOR = 3.9, 95%CI: 2.3–6.8), while working from entertainment venues (AOR = 0.3, 95%CI: 0.2–0.5) and public places (AOR = 0.2, 95%CI: 0.1–0.5) was associated with decreased awareness. Sex workers for whom conflict influenced entry into sex work had significantly lower odds of reporting awareness of NGOs compared to those whose entry into sex work was not influenced by conflict (AOR = 0.3, 95%CI: 0.2–0.8).

**Table 3:** Crude (OR) and adjusted odds ratios (AOR) with 95% confidence intervals (95%CI) from logistic regression models examining correlates of NGO awareness among female sex worker participants.

	**NGO Awareness**			
	**OR**	**95%CI**	**AOR**	**95%CI**
**Years in sex work**				
0–1	*Ref*.		-	-
2–4	2.37	(1.35, 4.16)	-	-
5–9	5.54	(3.16, 9.70)	-	-
≥10	4.84	(2.58, 9.08)	-	-
Education^*^				
Incomplete secondary education	*Ref*.	-	*Ref*.	-
Completed secondary education	0.83	(0.41, 1.67)	0.72	(0.34, 1.51)
Completed post-secondary education	1.16	(0.58, 2.30)	0.81	(0.39, 1.68)
Most common workplace^*^				
Office	*Ref*.	-	*Ref*.	-
Apartment	3.28	(1.94, 5.57)	3.93	(2.27, 6.82)
Entertainment venues	0.22	(0.13, 0.37)	0.28	(0.16, 0.48)
Public places	0.21	(0.10, 0.45)	0.24	(0.11, 0.53)
Highways or truck stops	0.96	(0.44, 2.12)	0.90	(0.40, 2.04)
Other	1.27	(0.50, 3.22)	1.43	(0.54, 3.80)
Conflict influenced entry into sex work^*^^†^
No	*Ref*.	-	*Ref*.	-
Yes	0.40	(0.18, 0.90)	0.35	(0.15, 0.82)
Recent migration^*^
No	*Ref*.	-	*Ref*.	-
Yes	0.68	(0.41, 1.12)	1.17	(0.67, 2.05)

* *Adjusted for years in sex work*.

† *n = 182; 364 respondents excluded due to entering sex work before conflict began; 13 respondents excluded due to incomplete data*.

### Factors Influencing HIV Testing in the Past 12 Months

As shown in Table 4, contact from an NGO outreach worker was associated with HIV testing in the past 12 months (AOR = 15.5, 95%CI: 8.5–28.2). As with NGO awareness, more years of experience in sex work was associated with increased testing in the past 12 months for those with 5–9 years (OR = 3.4, 95%CI: 2.0–6.0) and at least 10 years (OR = 2.1, 95%CI: 1.1–3.8) in the profession. Compared to those whose primary workplace was an office, all other sex workers reported lower rates of testing. Notably, women working in entertainment venues (AOR = 0.3, 95%CI: 0.2–0.5) and public places (AOR = 0.2, 95%CI: 0.1–0.3) were at significantly lower odds of reporting testing in the past 12 months. Accessing NGO services, such as receiving condoms (AOR = 16.9, 95%CI: 9.7–29.3), HIV informational materials (AOR = 13.3, 95%CI: 7.2–24.4), and HIV prevention counseling (AOR = 15.1, 95%CI: 6.9–33.3) were all associated with testing in the past 12 months. Sex workers whose entry into the profession was influenced by the conflict were at lower odds of testing for HIV (AOR = 0.4, 95%CI: 0.2–0.9).

**Table 4:** Crude (OR) and adjusted odds ratios (AOR) with 95% confidence intervals (95%CI) from logistic regression models examining correlates of HIV testing in the past 12 months among female sex worker participants.

	**Tested for HIV in the past 12 months**			
	**OR**	**95%CI**	**Adjusted OR**	**95%CI**
**Years in sex work**				
0–1	*Ref*.		*-*	-
2–4	1.31	(0.76, 2.25)	-	-
5–9	3.43	(1.97, 5.97)	-	-
≥10	2.09	(1.14, 3.83)	-	-
**Previous pregnancy**				
No	*Ref*.	-	-	-
Yes	1.63	(1.14, 2.33)	-	-
Education^*^				
Incomplete secondary	*Ref*.	-	*Ref*.	-
education				
Completed secondary	1.41	(0.70, 2.84)	1.30	(0.63, 2.67)
education				
Completed post-secondary	1.58	(0.80, 3.13)	1.23	(0.60, 2.50)
education				
Awareness of NGO and receipt of outreach services^*^				
Unaware	*Ref*.	-	*Ref*.	-
Aware with outreach	15.46	(8.47, 28.24)	12.96	(7.00, 23.97)
Aware without outreach	0.61	(0.38, 0.99)	0.50	(0.30, 0.84)
Most common workplace^*^				
Office	*Ref*.	-	*Ref*.	-
Apartment	0.80	(0.49, 1.30)	0.80	(0.48, 1.33)
Entertainment venues	0.25	(0.15, 0.42)	0.29	(0.17, 0.50)
Public place	0.14	(0.07, 0.30)	0.16	(0.07, 0.34)
Highways/truck stops	0.46	(0.20, 1.03)	0.42	(0.18, 0.96)
Other	0.24	(0.10, 0.59)	0.25	(0.10, 0.61)
Number of clients, past 30 days^*^				
0–19	*Ref*.	-	*Ref*.	-
20–29	1.52	(1.00, 2.31)	1.17	(0.75, 1.81)
≥30	1.63	(1.14, 2.33)	0.84	(0.514 1.38)
Recent migration^*^				
No	*Ref*.	-	*Ref*.	-
Yes	0.57	(0.34, 0.94)	0.77	(0.45, 1.33)
Conflict influenced entry into sex work^*^^†^				
No	*Ref*.	-	*Ref*.	-
Yes	0.43	(0.20, 0.94)	0.42	(0.19, 0.93)
Received condoms in past 6 months^*^				
No	*Ref*.	-	*Ref*.	-
Yes	18.15	(10.55, 31.23)	16.89	(9.74, 29.30)
Received HIV booklets/brochures in past 6 months^*^				
No	*Ref*.	-	*Ref*.	-
Yes	13.90	(7.64, 25.31)	13.28	(7.22, 24.43)
Received HIV prevention counseling in past 6 months^*^				
No	*Ref*.	-	*Ref*.	-
Yes	16.55	(7.56, 36.21)	15.14	(6.88, 33.31)

* *Adjusted for years in sex work and previous pregnancy*.

† *n = 182; 364 respondents excluded due to entering sex work before conflict began; 13 respondents excluded due to incomplete data*.

## Discussion

Supporting findings from earlier work in Ukraine (6, 31), our results suggest that NGOs in Dnipro play a large role in HIV testing and prevention, as a majority of participants reported receiving their most recent HIV test at an NGO, and most have received services from an NGO in the past 6 months. Prior to the full-scale war, Ukraine was in the process of implementing a healthcare reform agenda, in which the central government sought to take further responsibility for HIV testing and prevention (8). However, given the current context, the country's healthcare priorities and resource allocations will necessarily shift and plans for reform will inevitably be put on hold. Still, findings from our study highlight the importance of collaboration and continued engagement with NGOs to ensure a smooth transition once peace is restored, so that key populations continue to be able to access services in the spaces that best meet their needs.

Despite the critical positioning of NGOs in providing HIV services, over one-third (38%) of sex workers reported not being aware of these organizations, and an equal proportion (38%) had not received any services from an NGO in the last 6 months. These groups are overlapping but not equivalent; the nature of NGO outreach work in Ukraine has resulted in a subset of sex workers who reported having received a service from an NGO worker (e.g., receiving free condoms at their places of work) while not reporting awareness of any particular NGO, which may be explained with “office managers,” madams or other third parties being mediators between the social worker and a sex worker. Twenty-eight percent of sex workers reported neither awareness nor service use in the last 6 months.

Sex workers who most frequently worked in an office reported moderate levels of NGO awareness but high rates of testing. This may reflect ongoing testing services provided or encouraged by office managers. In other contexts, formal workplace typologies such as offices have been found to facilitate increased safety, improved agency to negotiate condom usage, and standardized protocols surrounding testing for sexually transmitted infections (32–34), suggesting that sex workers who work in these settings may have readily available access to testing services through their workplace. Entertainment venues and public places were both associated with low rates of awareness and testing, reflecting an opportunity for NGOs to focus on these venues to ensure access for new clients. No significant associations were observed for women who work at highways or truck stops, which may be due in part to the small subset of participants reporting this as their primary workplace (*n* = 30). Previous analyses have noted that sex workers in these workplaces tend to be older, see more clients, face higher rates of physical and sexual violence, and rate their personal safety as a sex worker as unsafe (35). This reflects increased vulnerability within this group and warrants further research into the unique dynamics of sex work at highways and truck stops.

Participants who had fewer years of experience in sex work reported lower testing rates and lower NGO awareness. These results are consistent with findings from a previous systematic review analyzing HIV testing behaviors among sex workers (36). Our findings suggest that once someone has been in sex work for 5 years or longer and still has not been connected to (or has chosen not to engage with) NGO services, then the likelihood that they will do so after that point is not significant. As such, while NGOs must focus on engaging with newer sex workers, simultaneously strengthening efforts to reach and engage sex workers who have been working for a longer period of time, but have not meaningfully engaged with prevention services, including HIV testing, will also be beneficial.

These data suggest that service providers offering HIV prevention services should consider strategies for broadening their reach to increase awareness of and access to services for sex workers who are currently not connected to prevention programs, including sex workers who have recently entered the profession and those working outside of “office” venues. Given our findings, NGOs or other service providing organizations could benefit from investing specifically in expansion of outreach networks for sex workers working in entertainment venues and public places, where NGO awareness was disproportionately low. Network- and enhanced peer-based approaches to promote service delivery have proven effective in increasing reach among those less connected to services (37). In addition, further work can be done to diversify service delivery platforms to accommodate the needs and preferences of potential service users.

Due to the structure of our questionnaire, data reflecting outreach worker contact was not collected for sex workers who reported being unaware of NGOs. As a result, those with outreach contact represented a subset of those who reported NGO awareness, even though this may not reflect reality. The strong effect of outreach may point to the direct effects of outreach on testing behaviors, although it is also possible that outreach contact serves as a proxy for other unmeasured variables associated with testing, such as involvement and interaction with NGOs. Notably, awareness was not associated with increased testing among women who did not receive outreach services. This importantly suggests that simply being aware of an NGO's existence is not sufficient to improve testing among sex workers in Dnipro; engagement with outreach services associated with an NGO is a necessary component to this process.

Based on these data, focusing efforts on expanding outreach workers' contact with sex workers could be an important strategy for increasing HIV testing uptake. This could be done most effectively by focusing on populations or locations with low levels of NGO awareness.

Among participants whose decision to enter sex work was influenced by the conflict, we observed reduced rates of both NGO awareness and HIV testing. This aligns with existing literature suggesting that conflict can negatively impact HIV-related health services (13–15). These observations might highlight a lack of opportunity to develop social and support networks that facilitate linkages to outreach and social workers among women whose entry into sex work was influenced by the conflict. Importantly, our findings suggest that in the context of conflict and other Big Events (38), NGOs and other organizations providing HIV prevention services should prioritize programming tailored for women who are newly entering sex work.

### Strengths and Limitations

This study has a number of strengths. First is the sampling framework, which used a comprehensive geographic mapping approach (17) to achieve a broad and representative sample of survey respondents. Additionally, the setting of Ukraine provides a unique context in which to study this topic against the backdrop of both national healthcare reform and ongoing armed conflict. Some limitations also exist due to the study's design. Data were collected through face-to-face surveys, which may have led to social desirability bias, and the cross-sectional nature of this study means that causality cannot be inferred. Furthermore, analyses presented in Table 4 do not include people who reported a previous positive HIV test but did not test in the past 12 months. Additional analyses revealed that only six respondents reported having a previous positive HIV test out of the 471 participants who had ever had an HIV test. Of those six participants, two had received a positive test in the past 12 months and were thus included in analyses. As years in sex work was determined to be an important confounder, we were only interested in the impact that years in sex work had on associations between independent variables and our two outcomes. Importantly, the quantitative nature of this study limits our ability to understand the processes underlying the observed associations. Qualitative research has been conducted as a part of the larger study (17) and will be presented in separate manuscripts. Finally, against the backdrop of war, the implications of these findings have inevitably shifted, and our recommendations will be most beneficial once Ukraine has returned to peacetime.

## Conclusions

NGOs play a significant role in HIV testing and prevention service provision, but a large portion of sex workers are not accessing services through these organizations. Increased awareness of and coverage of HIV testing and other prevention services among women who are new to sex work and those who work in hotspots we define as entertainment venues and public places should be prioritized by NGOs in Ukraine. Outreach workers play a critical role, as they provide HIV prevention information and supplies, as well as support linkages to HIV testing and treatment for sex workers. Once peaceful life resumes in Ukraine, continued support for NGOs and outreach workers must be considered as funding for HIV prevention services transitions to government-funding.

## Data Availability Statement

The datasets used and/or analyzed during the current study are available from the corresponding author on reasonable request.

## Ethics Statement

Ethical approval was obtained from the Health Research Ethics Board at the University of Manitoba, Canada [HS20653(H2017:097)]; the Ethical Review Committee of the Sociological Association of Ukraine, and the Committee on Medical Ethics of the L. Gromashevsky Institute of Epidemiology and Infectious Diseases at the National Academy of Medical Sciences of Ukraine, Kyiv. All participants provided written, informed consent. Participants 14 years of age were considered mature minors and provided consent to take part in the study without consent from their parent or guardian. This was approved by the local ethics committees.

## Author Contributions

MB, DP, OB, RL, MP, and SM conceived of the Dynamics Study objectives and design. OB, DP, and TT led study implementation. MB and SS conceived of the study objective for this paper and supervised data analysis. JD and AT analyzed the data. DP, LM, NH, and SS supported data analyses. JD wrote the first draft of the manuscript. LM, DP, NH, LL, SS, and MB developed, revised, and finalized the writing. All authors have read and approved the final version of the manuscript.

## Funding

This study was funded by the Canadian Institutes for Health Research [reference no. PJT-148876]. This funding source had no design in the study nor in the collection, analysis, or interpretation of the data. RL was supported by a Tier 2 Canada Research Chair in Global Intervention Politics and Social Transformation, SM was supported by a Tier 2 Canada Research Chair in Mathematical Modeling and Program Science, and SS was supported by a Tier 2 Canada Research Chair in Program Science and Global Health.

## Conflict of Interest

The authors declare that the research was conducted in the absence of any commercial or financial relationships that could be construed as a potential conflict of interest.

## Publisher's Note

All claims expressed in this article are solely those of the authors and do not necessarily represent those of their affiliated organizations, or those of the publisher, the editors and the reviewers. Any product that may be evaluated in this article, or claim that may be made by its manufacturer, is not guaranteed or endorsed by the publisher.
